# Development and prevention of ischemic contracture (“stone heart”) in the pig heart

**DOI:** 10.3389/fcvm.2023.1105257

**Published:** 2023-02-20

**Authors:** Mei Li, Zhi Qin, Erik Steen, Ann Terry, Bowen Wang, Björn Wohlfart, Stig Steen, Anders Arner

**Affiliations:** ^1^Department of Clinical Sciences, Lund, Lund University, Lund, Sweden; ^2^Igelösa Life Science AB, Lund, Sweden; ^3^MAX IV Laboratory, Lund University, Lund, Sweden

**Keywords:** stone heart, ischemic contracture, donation after circulatory death (DCD), Mavacamten, Ca2+-sensitivity, transplantation

## Abstract

Stone heart (ischemic contracture) is a rare and serious condition observed in the heart after periods of warm ischemia. The underlying mechanisms are largely unknown and treatment options are lacking. In view of the possibilities for cardiac donation after circulatory death (DCD), introducing risks for ischemic damage, we have investigated stone heart in pigs. Following cessation of ventilation, circulatory death (systolic pressure <8 mmHg) occurred within 13.1 ± 1.2 min; and a stone heart, manifested with asystole, increased left ventricular wall thickness and stiffness, established after a further 17 ± 6 min. Adenosine triphosphate and phosphocreatine levels decreased by about 50% in the stone heart. Electron microscopy showed deteriorated structure with contraction bands, Z-line streaming and swollen mitochondria. Synchrotron based small angle X-ray scattering of trabecular samples from stone hearts revealed attachment of myosin to actin, without volume changes in the sarcomeres. Ca^2+^ sensitivity, determined in permeabilized muscle, was increased in stone heart samples. An *in vitro* model for stone heart, using isolated trabecular muscle exposed to hypoxia/zero glucose, exhibited the main characteristics of stone heart in whole animals, with a fall in high-energy phosphates and development of muscle contracture. The stone heart condition *in vitro* was significantly attenuated by the myosin inhibitor MYK-461 (Mavacamten). In conclusion, the stone heart is a hypercontracted state associated with myosin binding to actin and increased Ca^2+^ sensitivity. The hypercontractile state, once developed, is poorly reversible. The myosin inhibitor MYK-461, which is clinically approved for other indications, could be a promising venue for prevention.

## 1. Introduction

Ischemic contracture of the heart (“stone heart”), is a rare but serious complication in cardiac surgery (1, 2). The condition develops as an irreversible contracture of the left ventricle, is resistant to several treatments and interventions, and often results in death of the patient. The critical initiating event is a period of warm ischemia where cellular contents of high energy phosphates decrease, followed by a Ca^2+^-insensitive myocardial contracture that severely increases coronary resistance and impairs perfusion, which makes pharmacological therapy complicated or impossible. The exact mechanisms underlying the contracture are still not known, they might include rigor cross-bridge attachment at low adenosine triphosphate (ATP) concentrations and possibly changes in cellular Ca^2+^ homeostasis (1, 3). Since possibilities to treat stone heart, once it has developed, are very limited or non-existing, modern cardiac surgeries introduce measures to limit periods with warm ischemia and attempt pretreatments with, e.g., Ca^2+^ antagonists, beta blockers (1, 3) and more recently with a sodium glucose cotransporter 2 (SGLT2) inhibitor empagliflozin (4).

Since the first successful cardiac transplantation in 1967 and the introduction of modern immunotherapy with cyclosporin in 1980s, heart transplantation is today performed successfully in several centers around the world. In Europe, more than 500 cardiac transplantations are performed annually, whilst more than 1,000 persons are on the active waiting list (Eurotransplant International Foundation, Retrieved September 2022).^1^ Thus, there is an urgent need to increase the number of suitable donor hearts, e.g., by improved care of the brain stem/brain dead donor (5) or using novel procedures to extend the time for transporting the donor organ (6). Since the number of suitable brain-dead donors (donation after brain death, DBD) is limited, approaches are made to also accept donors after circulatory death (donation after circulatory death, DCD), where the brain stem can be partially functional or after unexpected cardiac arrest (uncontrolled DCD). For DCD, legislation varies among countries and the time between cessation of circulation and organ procurement can vary (7). The DCD procedures will involve the risk for warm ischemia and stone heart. All techniques that cease the heart beating, i.e., cardioplegia (high K^+^ solutions) would minimize the cardiac work and prevent warm ischemia, but are not applicable due to ethical requirements for “hands off time” of at least several minutes after circulatory death in most countries.

Recent experimental studies have suggested that adequate control of blood pressure and heart rate can minimize the risk for stone heart development after withdrawal of life support (8). The idea is to minimize cardiac work. Mavacamten (MYK-461) is a small-molecule myosin inhibitor which acts at the myofilament level, and inhibits the force-generating myosins (9–11). It was recently approved by FDA to improve heart function in patients with obstructive hypertrophic cardiomyopathy (12). The therapeutic strategy was to normalize excessive contractility in the hypertrophic state (13). Since this compound enables a reduction of cardiac work, it could potentially provide an alternative approach to prevent stone heart, or delay its onset during warm ischemia and DCD.

We have developed a clinically relevant model for reproducible induction of the stone heart condition in pig, and characterized the development with cardiac ultrasound, ECG, and pressure recordings. The end stage, with clearly developed ischemic contracture, was examined using electron microscopy, small angle X-ray diffraction, biochemical determination of high energy phosphate compounds, and force recordings to determine Ca^2+^ sensitivity. Furthermore, an *in vitro* model with isolated trabeculae mimicking the process of the stone heart in whole animals was established, to examine potential approaches for prevention of the contracture. We have addressed the following questions in the pig model: (1) What is the time course for stone heart development after circulatory death? (2) Can the stone heart development be modeled in an *in vitro* system, for systematic investigation of key parameters and screening on potential treatments? (3) What is the extent of energy depletion in the stone heart and how are cellular structure and myofilament organization affected? (4) Is Ca^2+^ regulation of contraction affected? (5) Can the contracture development be attenuated by the myosin inhibitor (Mavacamten)?

## 2. Materials and methods

### 2.1. Animals, anesthesia, and physiological monitoring

Adult Swedish domestic pigs of both genders (males castrated after birth) weighing 40–50 kg (age approximately 90–120 days) were used. The animals were handled in compliance with the European Convention for the Protection of Vertebrate Animals Used for Experimental and Other Scientific Purposes (Directive 2010/63.EU). The experiments were approved by the local Animal Ethical Committee (5.8.18-15906/2020). All anesthesia and surgical procedures were performed by trained surgeons. The animals were anesthetized with an intramuscular injection of atropine 0.5 mg (Unimedic AB, Matfors, Sweden), xylazine 100 mg (Bayer, Solna, Sweden), and ketamine 20 mg/kg body weight (Intervet AB, Stockholm, Sweden) followed by an intravenous injection of fentanyl 4 μg/kg (Braun, Melsungen, Germany) and midazolam 0.4 mg/kg (Hameln Pharma Plus GmbH). A catheter was placed in an ear vein and the anesthesia was maintained with an intravenous infusion of ketamine [10 mg/(kg × h)] and rocuronium bromide [1.5 mg/(kg × h)], (Fresenius Kabi Austria GmbH, Graz, Austria). Animals were tracheostomized and connected to a respirator with volume-controlled and pressure-regulated ventilation. Catheters were placed in the carotid artery (advanced to the aortic arch) and *via* the carotid vein to the right atrium for determination of arterial and venous pressures. ECG was monitored using electrodes on the chest. Pressures and ECG signals were recorded using amplifier systems and AD converters from AD Instruments, evaluated using LabChart program (AD Instruments, Sydney, NSW, Australia). Ultrasound imaging (M-mode) of the heart was performed using a Siemens Acuson Sequoia 512 system with 8V5 probe (8.5 MHz). The heart was scanned in the midline of the left ventricle, enabling measurements of left ventricular wall thickness and calculation of fractional shortening from the percentage change in left ventricular diameter during systole. The chest cavity was opened and the pericardium was cut open for access of the ultrasound probe. The heart was covered with damp cloth to prevent drying.

### 2.2. Stone heart in anesthetized pigs

Asphyxia was initiated (time = 0) by clamping the tracheal tube. The cardiac parameters (pressures, ECG) were continuously monitored and ultrasound recordings were made at regular intervals. The time point when systolic pressure had fallen to 40 mmHg, and to 8 mmHg (a situation where no circulation occurs) were noted. The stone heart (ischemic contracture) state was determined by manual palpation of the left ventricular stiffness and confirmed with ultrasound (asystole with left ventricular wall thickness increased more than 50%). These examinations were performed every 5 min. When the stone heart state was reached, samples were taken for ATP/phosphocreatine (PCr) determination, preparation of skinned fibers, and fixation for electron microscopy (see below). These samples were compared with samples obtained from fresh control hearts in separate experiments on anesthetized animals. Photographs were taken of the normal heart and the stone heart condition during the operative procedures, and of slices from the mid-section of the hearts. The slices were stained with 1% TTC (triphenyl tetrazolium chloride, Sigma-Aldrich, Darmstadt, Germany) to identify necrotic regions. In brief, slices were immersed in 1% TTC, and incubated at 37°C for 30 min. The samples from hearts with stone heart condition were compared with samples of fresh control hearts obtained from anesthetized animals.

### 2.3. ATP/PCr determination

Myocardium (from whole hearts) and trabecular samples (from *in vitro* experiments) were rapidly frozen with Wollenberger clamps precooled in liquid nitrogen, and stored at −80°C until analysis. The samples were weighed, crushed, homogenized in perchloric acid, centrifuged and neutralized with KOH. Aliquots of the homogenate were then assayed for ATP and PCr using an NADPH linked assay as described by Passonneau and Lowry (14). The fluorescence signal of samples in triplicate (excitation 355 nm, emission 460 nm) was measured using a FLUOstar Omega Microplate Reader (BMG LABTECH GmbH). Standards of ATP and PCr were analyzed in parallel. The samples were analyzed blinded and the contents of the high energy phosphates are given relative to sample wet weight.

### 2.4. Transmission electron microscopy

Thin strips of left ventricular myocardium were dissected from fresh control and stone hearts. The samples were gently stretched to about 1.2–1.3 × slack length (corresponding to approximate optimal length for force development), and fixed in 2% paraformaldehyde and 2.5% glutaraldehyde in 0.1 M cacodylic buffer (pH 7.4) for 24 h at 4°C, followed by post-fixation in 1% osmium tetroxide in 0.1 M cacodylic buffer for 2 h at 4°C. The samples were dehydrated and embedded in epoxy resin (Agar 100). Semi thin-sections (1.5 μm) were stained with Richardson’s solution to examine the orientation of the tissue block. Ultra-thin sections (50 nm) were stained with uranyl acetate and lead citrate, and examined using a JEOL JEM 1400 Plus transmission electron microscope.

### 2.5. Skinned fiber preparations and calcium sensitivity determination

Thin bundles of the left ventricular wall were teased/cut out and chemically skinned using a protocol described by Lu et al. (15). The samples were stored in 50% glycerol at −20°C until analysis. Thin preparations (diameter of about 200 μm, length about 1 mm) were prepared and glued using cellulose acetate glue between two carbon pins, one attached to a micrometer screw for length adjustment, and the other to a AE801 force transducer (Kronex, Oakland, CA, USA). The samples were stretched to about 1.3 × slack length, and kept in 0.5 ml Perspex baths at 22°C. Solutions and procedures were as described previously (16). After 30 min in relaxing [pCa = −log_10_([Ca^2+^]) 9] solution with 1% Triton X-100 and a further brief washout in relaxing solution, the preparations were activated at pCa 4.7 to determine initial maximal force responses. Thereafter, the muscles were relaxed (pCa 9) to determine residual tension and activated at increasing Ca^2+^ levels (pCa 6.6, 6.0, 5.7, and 4.7). The force at each Ca^2+^ level was normalized to the initial maximal force response. The force (*F*) and [Ca^2+^] (C) data of each sample was analyzed by fitting a hyperbolic function [*F* = *R* + *M* × C*^h^*/(C*^h^* + EC_50_*^h^*)], where the fitted parameter *R* corresponds to the residual tension at pCa 9, *M* the maximal response, *EC*_50_ the concentration giving half-maximal tension and *h* the Hill steepness coefficient.

To examine the effects of the Mavacamten (MYK-461), skinned fibers were activated at pCa 4.7 and exposed to increasing concentrations of MYK-461. Composition of the solutions for skinned fibers were calculated as described by Fabiato (17). MYK-461 was purchased from Selleckchem (Planegg, Germany), and dissolved in DMSO.

### 2.6. Ischemia/hypoxia *in vitro*

Fresh control hearts were perfused with cardioplegic solution (Plegisol^®^, Medline, Arnhem, Holland, modified with increased KCl to 23 mM) in anesthetized animals. The hearts were exercised and cooled (4°C) in cold cardioplegia for 30 min before further preparation. Trabecular muscles, free from side branches, were identified in the right ventricular wall, and carefully dissected. Sutures (6/0 silk) were tied at each end, and the preparations were transferred to 50 ml open organ glass baths filled with Krebs’ solution at 37°C gassed with 95/5% O_2_/CO_2_ giving a pH of 7.4. The muscles were mounted vertically, with one end attached to a fixed pin in the bath, and the other connected to a Grass FT03 force transducer. Stimulation was applied using a Grass S48 stimulator at 0.5 Hz, 0.5 ms pulse duration and supramaximal voltage. The muscles were allowed to accommodate for about 20 min. When stable contractions could be elicited, the preparations were stretched to a length giving maximal force (about 1.3 × slack length). Initial maximal force responses were recorded on all preparations. The trabecular preparations varied in absolute active force generation between 3 and 10 mN depending on size. Preparations giving less than 3 mN or having increased basal tone during accommodation were excluded. Ischemia/hypoxia was induced by transfer to Krebs’ solution made without glucose, and with the bath gassed with 95/5% N_2_/CO_2_. Using a polarographic O_2_ electrode (MLT1120, ADInstruments), the O_2_ pressure in the bath was monitored. With N_2_/CO_2_ gassing, PO_2_ reached below 7 mmHg in about 4 min. The ischemia/hypoxia was maintained for 30 min during which stimulation was continued. Thereafter, normal Krebs’ solution and O_2_ were reintroduced, and the recovery phase of the muscles was followed for 30 min. Samples for ATP/PCr measurements were taken after accommodation and at the end of the ischemia/hypoxia period.

Different solutions were examined during the hypoxia period: glucose-free Krebs’, modified Plegisol^®^, glucose-free Krebs’ with 23 mM KCl or 16 mM MgCl_2_. To examine the effect of MYK-461, the compound was introduced 20 min prior to, and maintained during the hypoxia/ischemia period in the Krebs’ solution. DMSO (<0.1%) was used as a solvent control for the MYK-461 experiments.

### 2.7. Small angle ***X***-ray diffraction

To determined possible effects on myofilament organization by the stone heart development, trabecular preparations were isolated from fresh control hearts and from hearts with developed stone heart condition. The muscle strips were mounted horizontally using silk thread in a temperature controlled (37°C) cuvette in MOPS buffered physiological solution (NaCl 118, KCl 5, Na_2_HPO_4_ 1.2, MgC_2_ 1.2, CaCl_2_ 1.6, glucose 10 mM, pH 7.4). The solution was gassed with air and exchanged every 5–10 min. The cuvette was equipped with Kapton windows and mounted on a stand in the CoSAXS beamline at the MAX IV synchrotron light facility, Lund, Sweden. The X-ray beam (wavelength 1 Å), had a size at the sample of about 50 × 60 μm. The sample detector distance was set to 3.5 m which gave a good resolution of the equatorial pattern using exposures of 0.5–1 s. The sample was moved between exposures to prevent beam damage. Scattering patterns were recorded using an EIGER 2 × 4 M detector (Dectris AG, Baden-Daettwil, Switzerland) and analyzed using a dedicated software. For each sample, recordings were made at different lengths (*L*) starting at slack length (*L*_*s*_) and stretched to 1.0, 1.2, 1.3, and 1.4 *L*/*L*_*s*_. At each length, the filament lattice spacing and intensity of the equatorial reflections were determined.

### 2.8. Statistical analysis

All data are presented as mean ± SEM. *N*-values refer to number of trabeculae or pigs as indicated. Statistical analyses and graphs were made using GraphPad Prism (Version 9.0) or SigmaPlot 14 for Windows. Student’s *t*-test was used when two groups were compared and repeated measures ANOVA was used for data including multiple measurements. Curve fitting was performed using non-linear and linear regression techniques implemented in the software.

## 3. Results

This study is based on demonstration of stone hearts (ischemic contracture) in comparison to fresh hearts in anesthetized animals. These results are combined with a newly developed *in vitro* model for ischemic contracture.

### 3.1. Stone heart development in anesthetized pigs

**FIGURE 1:**
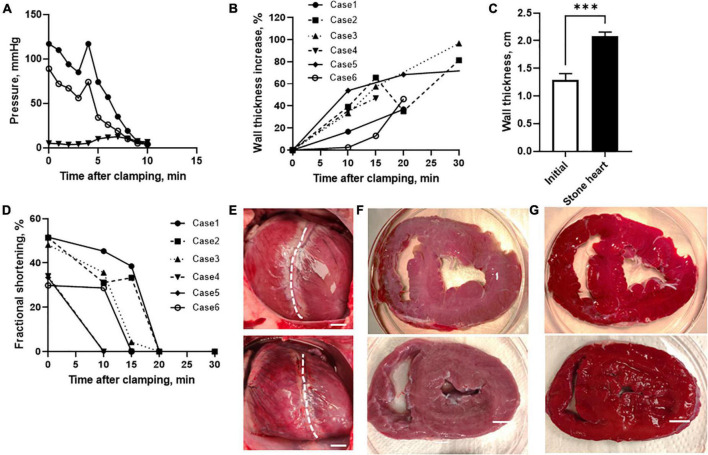
Development of stone heart in a pig model. **(A)** Systolic (filled circles), diastolic (open circles), and mean venous (triangles) pressure of a pig after induction of asphyxia. **(B)** Left ventricular wall thickness (diastolic) relative to initial at different time after trachea clamping, determined using ultrasound in individual animals (case 1–6). Note, case 5 reached stone heart at 60 min after asphyxia. **(C)** Mean left ventricular wall thickness before asphyxia and in the stone heart condition (*n* = 6 in animals each group). **(D)** Fractional shortening determined at different time points using ultrasound. **(E)** Photos of hearts prior to asphyxia (upper) and after development of ischemic contracture (stone heart condition, lower), dashed line indicates the border between left and right ventricles, with left ventricle is oriented to the right. Note, the enlarged right ventricle (due to pooling of blood) and the contracted left ventricle in the stone heart condition representative photos in the series of six animals. **(F)** Slices through the mid-section of a normal heart (upper) and a stone heart (lower). **(G)** Corresponding TTC stained slices. Representative photos in the series of four animals in each group. Scale bars in **(E–G)**: 1 cm. ^***^p < 0.001.

To model the stone heart development *in vivo*, we performed experiments in anesthetized pigs. Figure 1A shows original traces of systolic, diastolic arterial and mean venous pressures following clamping of the trachea (time = 0). The asphyxia resulted in a gradual fall in arterial pressure. After 3 min, gasping occurred in this animal, which gave a transient increase in arterial pressure. Thereafter, arterial pressure fell, and was at 40 mmHg at about 6–7 min, and at 8 mmHg after about 9 min, a situation when the arterial pressure equals the venous pressure (i.e., circulatory death) and no circulation occurs. The average time to reach 40 and 8 mmHg after initiation of asphyxia was 10.2 ± 1.3 and 13.1 ± 1.2 min (*n* = 6 animals), respectively. ECG and pressure monitoring showed a transient increase in heart rate during the gasping phase, and variable arrhythmia patterns with supraventricular and ventricular extra systoles, prolonged P-Q times, mechanical *alternans* and, in some cases, ventricular fibrillation at the end. In all six examined animals, a clear stone heart (ischemic contracture) was developed within 15–60 min after asphyxia. Average time from reaching 8 mmHg to stone heart was 17 ± 6 min (*n* = 6 animals). This was demonstrated using ultrasound examination (M-mode) as a statistically increased wall thickness (Figures 1B, C) and a gradual decrease in fractional shorting of the left ventricle toward asystole (Figure 1D). Figures 1A, B, D show behavior of individual experimental animals. The stone heart samples were obtained in the situation where beating had ceased, and the wall thickness had increased by more than 50%. This stage was also examined by manual palpation revealing a stiff structure and a super-contracted left ventricle, as seen in the photographs in Figures 1E, F. Figure 1G shows photographs of heart sections stained with 1% TTC where red areas indicate viable myocardium. No visible signs of necrosis (white staining) were noted.

### 3.2. Structural examination of the stone heart

**FIGURE 2:**
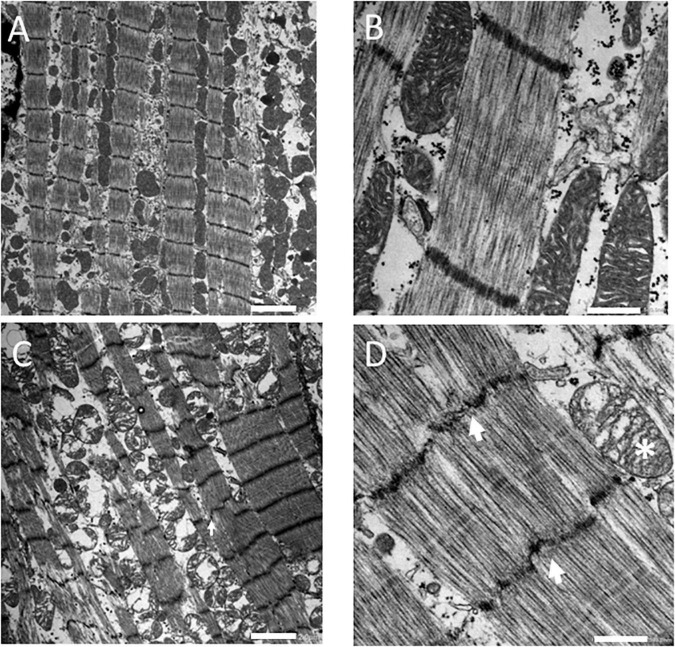
Electron microscopy images of fresh control **(A,B)** and stone heart **(C,D)** samples. Scale bar: 2 μm **(A,C)**, 0.5 μm **(B,D)**. Arrows indicate Z-line streaming, asterisk indicates a swollen mitochondrion. Electron microscopy was performed on two animals in each group.

Figure 2 shows electron microscopy images of the myocardium. The control samples (Figures 2A, B) were characterized by regular sarcomere patterns in myofibrils with well-defined Z-lines surrounded by rows of intact mitochondria. In contrast, the samples from stone hearts (Figures 2C, D) showed disorganized sarcomeres, Z-line irregularities and swollen mitochondria.

**FIGURE 3:**
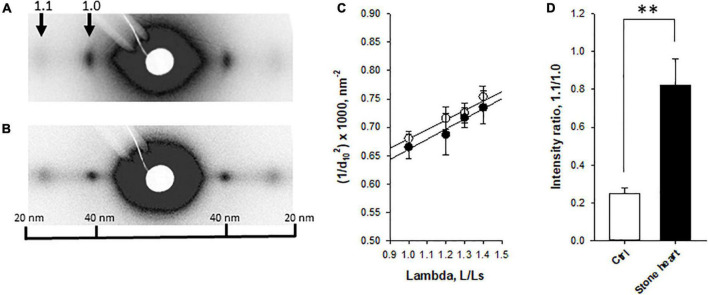
Small angle X-ray diffraction of fresh control and stone heart samples. Panels **(A,B)** show original recordings from a control **(A)** and a stone heart **(B)**, with equatorial 1.1 and 1.0 reflections indicated. Panel **(C)** shows the spacing of the 1.0 reflection (*d*_10_) plotted as 1/*d*_10_^2^ at different lengths (*L*) relative to slack length (*L*_*s*_). Straight lines were fitted to the relations. Panel **(D)** shows average 1.1/1.0 intensity at optimal stretch. Filled symbols and filled bars, stone heart; open symbols and bars, fresh hearts. *N* = 6–7 trabeculae in each group (from two animals in each group). ^**^p < 0.01.

To examine if the stone heart condition was associated with changes in filament distances (shrinkage or swelling of the lattice) or mass transfer of myosin heads toward the thin filaments, we performed small angle X-ray diffraction experiments on trabecular strips obtained from fresh control and stone hearts. The equatorial patterns were clearly resolved (Figures 3A, B) in both groups. No difference in 1.0 spacing (*d*_1.0_) was observed between the groups at optimal length (control 37.6 ± 0.5 nm, *n* = 7, stone heart 37.4 ± 0.5 nm, *n* = 6 trabeculae, from two animals in each group). Figure 3C shows a plot of 1/*d*_1.0_^2^ at different lengths (*L*) relative to slack (*L*_*s*_). The straight line suggests a constant volume relationship with shrinkage of the lattice spacing as the muscle is stretched, and that no differences in sarcomere volume are observed between the two groups. The range of sarcomere length that could be investigated is, however, limited and laser diffraction measurements of sarcomere length could not be done with accuracy over the whole stretch interval. It is thus difficult to calculate the sarcomere volume exactly (18), but if we assume a sarcomere length of 2.2 μm at optimal stretch, 1.3 *L*/*L*_*s*_ (19), the volume would be about 3.5 × 10^–3^ μm^3^ in both control and stone heart. This is close to the value reported by Matsubara and Millman (20) for cat heart muscle (3.0 × 10^–3^ μm^3^). As seen in Figures 3A, B, D the 1.1/1.0 intensity ratio at optimal length was significantly increased in the stone heart group. This parameter would increase upon mass transfer of the myosin heads toward the thin filaments.

### 3.3. High-energy phosphates in the stone heart

**FIGURE 4:**
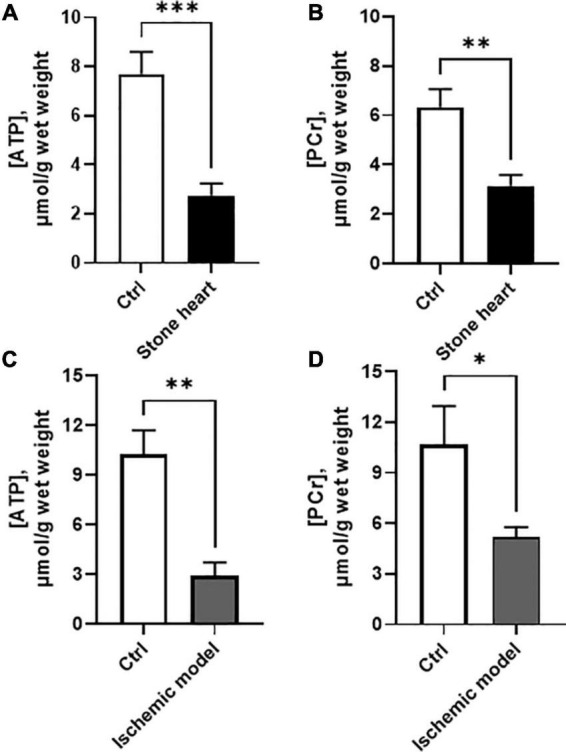
ATP and PCr contents in samples taken from the hearts of anesthetized pigs **(A,B)** and trabeculae *in vitro* **(C,D)**. Controls (beating hearts or oxygenated trabecular preparations) are shown with open bars, and stone heart/ischemia *in vitro* with filled bars. *N* = 3–10 in each group (from two to four animals in each group). *p < 0.05; ^**^p < 0.01; ^***^p < 0.001.

Figures 4A, B show contents of high energy phosphates (ATP and PCr) in rapidly frozen samples of the left ventricular myocardium from control and stone hearts. The stone heart condition was associated with significant decreases of both ATP and PCr. The levels of ADP contents are difficult to measure and estimate; several factors have to be considered: potential contamination from structural ADP, ADP binding to cellular proteins, [H^+^] changes and ADP distribution in the cell, e.g., in mitochondria (21). We can therefore not give an estimate of the ADP increase.

### 3.4. Calcium regulation of contraction in permeabilized trabecular muscle

**FIGURE 5:**
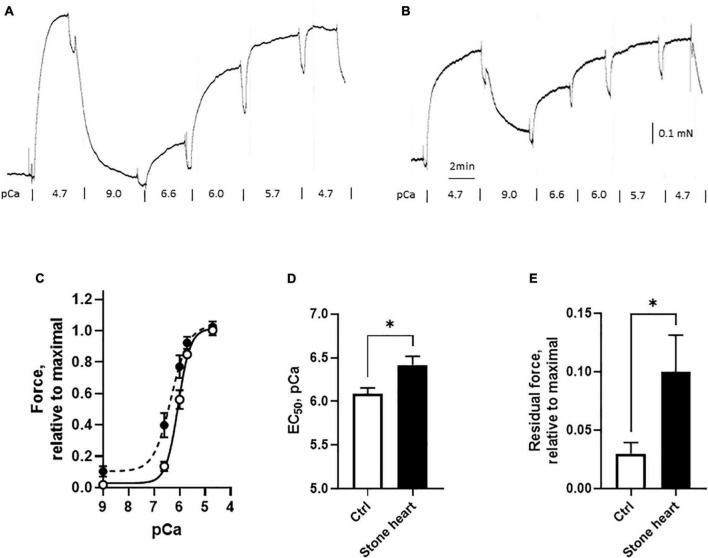
Calcium sensitivity of skinned cardiac fibers from control (open symbols and bars) and stone hearts (filled symbols and bars). Original traces of contractile responses in a control fiber **(A)** and a stone heart fiber **(B)**. Force, relative to initial maximal response vs. pCa **(C)**. Summarized data showing EC_50_ in pCa units **(D)** and the residual force at pCa = 9.0 **(E)**. *N* = 11–12 preparations in each group (from four animals in each group). *p < 0.05.

To investigate the regulation of the contractile machinery, we performed experiments on chemically permeabilized trabecular preparations from the left ventricle of control and stone hearts. The preparations were mounted in the relaxed state, contracted to obtain an initial maximal response (pCa = 4.7) and then relaxed (pCa = 9.0) prior to the accumulative addition of the Ca^2+^ steps. The responses to different free Ca^2+^ concentrations were measured to determine the calcium sensitivity relationship (Figure 5C). These experiments were performed to examine Ca^2+^ sensitivity and the absolute stress levels in the two groups would not affect the results. However, the preparations were made with similar size (diameter about 200 μm) and if active stress was estimated, it was 22 ± 3 vs. 18 ± 5 mN/mm^2^ (*n* = 11–12 trabeculae in each group, four animals in each group) in controls and stone hearts respectively. This excludes major differences in active force generation between the two groups. As seen in the original recordings of Figures 5A, B, and in the summarized data of Figures 5C, D, the stone heart samples exhibited a leftward shift with a significant decrease in the EC_50_ value (i.e., increased pCa value) for the responses to calcium. The final response to maximum calcium concentration (pCa = 4.7) at the end of the calcium dose response curve was similar to the initial, confirming that the preparations did not deteriorate during the experiments. However, the residual force level in the relaxed state (pCa = 9.0) after the initial contraction was higher in the stone hearts (Figure 5E), also seen in the original trace of Figure 5B, suggesting a component of calcium-independent contraction. Evidently, this is not prominent prior to the first Ca^2+^ activation, which might imply that it is due to an impaired relaxation.

### 3.5. Ischemic contracture *in vitro*

**FIGURE 6:**
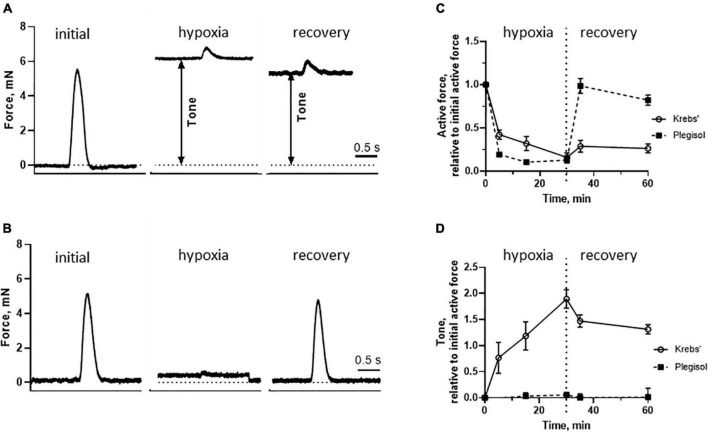
*In vitro* force measurements from paced intact trabecular muscles. Original force trace of a single contraction prior to hypoxia/ischemia, at the end of a hypoxia period and at the end of a recovery period (left to right). The preparation in panel **(A)** was treated with glucose free Krebs’ solution and the preparation in panel **(B)** with cardioplegic solution (Plegisol^®^) during the hypoxia/ischemia period. Initial and recovery periods were both recorded in Krebs’ solution. Summarized data of active force **(C)** and tone **(D)** in each group at different times. Hypoxia/ischemia was introduced between 0 and 30 min, followed by recovery for additional 30 min. The Plegisol^®^ group had significantly lower tone during both the hypoxia/ischemia and recovery periods, and higher active force during recovery (*p* < 0.05 for both comparisons, repeated measures, ANOVA). *N* = 4–8 trabeculae in each group (from six animals in each group).

To develop an *in vitro* model for stone heart, we isolated trabecular preparations from fresh control hearts and examined in open organ baths. From each heart, four samples were mounted, contracted by electrical stimulation and investigated in parallel. The preparations were stretched and analyzed at optimal length (about 1.3 *L*/*L*_*s*_). Using this setup, we have established a reproducible *in vitro* model for examining the responses to hypoxia/ischemia under controlled conditions. Ischemia was mimicked by using glucose-free Krebs’ solution. An advantage is that samples from the same heart can be analyzed in parallel, with one group exposed to hypoxia/ischemia, while the other served as control. The challenge with low oxygen and in the absence of glucose in the Krebs’ solution resulted in a drop in active tension during a 30-min observation period. The active force response dropped to about 20% of the initial value after 30 min (Figures 6A, C). At the same time, a sustained tonic force (contracture) developed reaching about 2 times of the initial active force level (Figures 6A, D). Re-oxygenation and introduction of glucose for 30 min did not result in a significant recovery of active force (Figure 6C) or a decrease in the tone (Figure 6D).

The ATP and PCr levels in the isolated trabecular preparations (Figures 4C, D) under oxygenated conditions in the presence of glucose were slightly higher than those obtained in the beating heart *in vivo*, although no significant differences could be detected between these two models. The hypoxia/ischemia of 30 min resulted in a significant drop in both ATP and PCr, in a similar manner as in the hearts of anesthetized animals.

### 3.6. Reversal and prevention of stone heart development *in vitro*

In the current *in vitro* model, we were not able to reverse the ischemic contracture at the end of the recovery period with BDM (10 mM), cardioplegic solution (modified Plegisol^®^) or the myosin inhibitor MYK-461 (1 μM), suggesting that the contraction, once developed, is very difficult to reverse. Instead, we introduced potential prevention strategies before/during the hypoxia/ischemia period. Figures 6B–D shows the effects of cardioplegic solution (modified Plegisol^®^) which promptly inhibited the electrically induced contractile responses and the ischemic contracture. Moreover, the recovery after the hypoxia/ischemia period under these conditions was rapid with normal contractions and absence of the residual tone. These results suggest that the inhibition of active contractions and reduced work performance during warm ischemia/hypoxia period can prevent the contracture development. The cardioplegic solution (modified Plegisol^®^) contains 16 mM MgCl_2_ and 23 mM KCl to ensure full inhibition of contraction (22). Interestingly, we found that treatment with high Mg^2+^ (16 mM MgCl_2_) alone in normal Krebs’ solution was sufficient to inhibit active contraction and prevent the ischemic contracture. After washout, the impaired active force was recovered to about 80% (*n* = 2) of initial value; and the contracture was about 10%. Similar beneficial effects were seen in Krebs’ containing high K^+^ (23 mM KCl), albeit the contracture was slightly larger (about 20%, *n* = 2).

**FIGURE 7:**
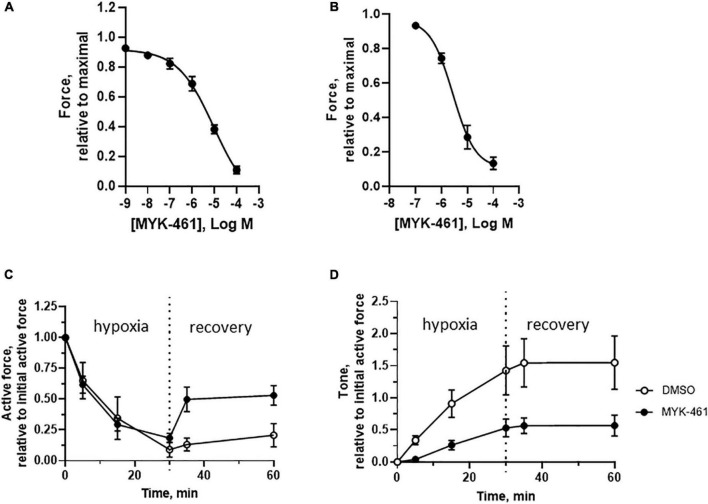
*In vitro* effects of MYK-461 (Mavacamten) on cardiac muscles. **(A,B)** Inhibitory effect on active contraction of MYK-461 in intact trabecular (**A**, EC_50_ = 9.3 μM) and in skinned cardiac fibers at pCa 4.7 (**B**, EC_50_ = 2.8 μM). Summarized data of active force **(C)** and tone **(D)** in non-treated controls and preparations incubated with 1 μM MYK-461 (initial time = 0, hypoxia/ischemia time = 0–30 min, recovery time = 30–60 min). MYK-461 was introduced 20 min prior to hypoxia/ischemia, and maintained during the challenge period. The MYK-461 group had significantly lower tone during both the hypoxia/ischemia and recovery periods, and higher active force during recovery (*p* < 0.05 for both comparisons, repeated measures, ANOVA). *N* = 4–8 trabeculae in each group (from four to six animals in each group).

We applied a cardiac myosin inhibitor to prevent the unwanted contraction and thereby preserve energy levels in muscle cells during hypoxia. We examined the inhibitory effects of the myosin inhibitor Mavacamten (MYK-461) in both isolated trabecula (Figure 7A) and skinned fibers (Figure 7B). These data show a significant reduction of active force with an EC_50_ value of about 3 μM. However, the inhibition of the highest dose of MYK-461 in skinned or intact fibers could not be reversed within 1 h after washout. In the isolated intact trabeculae, we applied a treatment period (30 min incubation prior to and during ischemia/hypoxia) with 1 μM MYK-461. This concentration is similar to the target plasma concentration in clinical studies (23), and gives about 25% inhibition of active contraction in our setup. After incubation of MYK-461 during the ischemia challenge, the recovery of active tension was significantly improved compared to controls (Figure 7C) reaching about 50% of the initial force. At the same time, the ischemic contracture development (tone) was significantly reduced (Figure 7D) and was 50% of the initial active force compared to the 150% in the non-treated control group.

## 4. Discussion

We show that the time course of stone heart development *in vivo* can vary between individuals, but the end stage, characterized by asystole, hypercontracted left ventricle is similar. The development of stone heart is most likely inhomogeneous with apex first affected early in the process. The pig heart is in many aspects very similar to the human heart, and the structural changes in the stone heart condition are similar. The time course might differ between species and be affected by, e.g., age, gender, underlying disease, and other factors. We have, however, focused on the fully developed stone heart in the pig, engaging the whole left ventricle. It is evident that the stone heart development is dependent on lack of energy in the myocardium. This is consistent with previous reports using rodent models where high glycogen content and or insulin can delay the onset of ischemic contracture and prevent injury (4, 24). The variation in time course for stone heart development in our pig model could reflect the contractile activity during the ischemia period where development of arrhythmia might affect the energy consumption. The possibility of additional oxygen delivery during gasping further can delay the onset of the stone heart. Our measurements of ATP and PCr contents show a reduction in these two high-energy phosphates. The reduction magnitude is smaller than what has been reported for an ischemic dog model (25), nevertheless, a lower drop in ATP has also been found in ischemic heart of the dog (26). The comparisons between the stone heart condition, and ischemia or hypoxia models are difficult because the development of the stone heart is not primarily ischemic and the decay of oxygen delivery is gradual. The drop in both ATP and PCr indicates an increase in ADP, although it is difficult to estimate. The challenge of low energy contents to the heart in stone heart condition is associated with structural changes in the contractile system with hyper contraction and misalignment of the myofilaments. These findings suggest irreversible modification of the filament lattice, e.g., *via* activation of proteolytic pathways, pH effects or protein oxidation. It is possible that the lack of energy results in calcium overload and activation of proteolytic pathways or that mitochondrial dysfunction leads to release of oxidative compounds (27). An important aspect is the deterioration of mitochondrial structure as shown by the electron microscopy. These alterations are most likely not reversible within the examined time scale, and could contribute to the irreversibility of the stone heart.

Structural changes in skeletal muscles *post mortem* include a drop in pH, and rigor development leading to irreversible consequences with loss of contractile function and shrinkage of the lattice spacing (16). The cardiac muscles during stone heart development lost the ability to generate the electrically induced active contractions, although the maximal force generating capacity, as judged from the magnitude of the contracture is maintained. It is difficult to determine the origin of the contracture, which is not reversed by relaxant factors (cardioplegia) or myosin inhibitors (MYK-461 or BDM). It is not due to shrinkage of the cells since X-ray diffraction did not reveal any decrease of the lattice volume. The mass transfer of myosin toward the thin filament, suggest however that the fully developed stone heart includes a component of force generating/maintaining cross-bridge attachment.

A key feature of the stone heart is the contracture development which suggests an activation of excessive contraction or rigor formation. The first possibility can be due to calcium overload or increased calcium sensitivity. Our data from skinned fibers suggest that an increased sensitivity to calcium is an important contributing factor to the contractile activation which in turn would increase the energy consumption. It should be noted that this mechanism does not exclude changes upstream of the Ca^2+^ regulation. Alterations in free Ca^2+^ seem to be excluded by data on ischemic cardiomyocytes (28, 29), but several factors including increases in [H^+^] (30) can be involved. The mechanism for the increased Ca^2+^ sensitivity can originate in the thin filament regulatory system, where changes in the troponin structure can alter the myofilament sensitivity to calcium (31, 32). In addition, attachments of non-regulated myosin heads as suggested by the X-ray scattering data, could lead to increased Ca^2+^ sensitivity due to cooperative effect on the thin filaments enhanced at low MgATP (33, 34). A population of unregulated myosin acting as rigor-like heads can lead to contractile activation, but their number would be quite low since the skinned fibers where ATP supply is not limiting, can relax significantly. Once the ischemic contracture is developed, it is not simply inhibited by several contractile inhibitors, suggesting that the ischemic contracture converts to a rigor-like contraction, independent of calcium, toward the end of stone heart development.

A rigor force would be inhibited by ATP, but this cellular component is difficult to increase *in vivo* since the mitochondrial system is affected. However, the ATP level, although significantly decreased, is still in the millimolar range in the stone heart. Studies in permeabilized, skinned, cardiac muscle show that rigor is developed when ATP concentration is in the range 0–100 μM which is significantly lower than our measured values (35). It is thus unlikely that the contracture reflects solely a rigor tone.

Once the stone heart is developed it seems difficult to reverse, as shown by the *in vitro* data. Also, administration of drugs would be difficult due to the high wall tension and poor coronary circulation. Thus, preventive measures, possibly introduced after withdrawal of life support in DCD would be relevant. Small molecule myosin inhibitors have recently been introduced on the basis of studies of cells and skinned fibers from human hearts and has reached clinical trials for treatment of hypercontractile states of the heart (13). In particular, the compound Mavacamten (MYK-461) has been examined and applied for treatment of hypertrophic cardiomyopathy, and could be a suitable candidate for preventing stone heart. Since information on intact preparations and skinned pig fibers is limited, we examined the effects on the pig heart samples and demonstrated a significant reduction in active force using MYK-461, in concentrations that can be achieved in clinical studies in humans. Although the compound inhibited the contraction of the trabeculae, it did not abolish the contractions completely. It might therefore, in view of the clinical approval for other conditions, be considered as a preventive treatment for ischemic injury introduced during the time between withdrawal of life support and procurement of the heart (hands-off time) in DCD.

Some limitations of the study can be noted. The results regarding protective effects of high Mg^2+^ and high K^+^ are based on few experiments, but are included as preliminary observation. We focus on a defined end-stage stone heart. The time course of the stone heart development *in vivo* shows some variation where the underlying mechanisms could be further explored. The protective effects of MYK-461 require additional investigation prior to a potential clinical application in the future.

## 5. Conclusion

In summary, the time course of stone heart development varies between individuals, and is shortened if specific arrhythmias (e.g., ventricular fibrillation) occur, and is slightly prolonged with gasping. The end stage is manifested by a hypercontracted state and is initiated by loss of mitochondrial function and ATP generation. The contracture is associated with an increased calcium sensitivity, possibly potentiated by unregulated attachments of myosin heads. The contractile state, once developed, is poorly reversible *in vivo*, most likely due to destruction of the cellular energy production and irreversible changes in the contractile structure and regulation. Since the ischemic contracture is essentially irreversible and most likely reflects a rigor-like state, clinical efforts should be made to prevent the initiation of the stone heart. Cardioplegia appears to be very efficient in this context, most likely by the significant inhibition of contractile activity and reducing the energy consumption. However, from clinical and ethical perspectives, it is not applicable since the administration of cardioplegia to a patient would result in direct termination of cardiac function. The myosin inhibitor MYK-461, which is clinically approved for other indications (cardiac hypertrophy), could be a promising venue. Since the effects on active contraction are not significant in the lower dose range, and treatment with MYK-461 has significant effects on the stone heart development in the pig model. The mechanism is unclear, but most likely partial inhibition of myosin activation results in a lower energy consumption and a slower development of stone heart.

## Data availability statement

The original contributions presented in this study are included in the article/Supplementary material, further inquiries can be directed to the corresponding author.

## Ethics statement

This animal study was reviewed and approved by the Lund Animal Ethical Committee (5.8.18-15906/2020).

## Author contributions

ML, SS, and AA conceived and designed the study, performed the experiments and data analyses, and wrote the manuscript. ZQ, ES, AT, BWa, and BWo performed the experiments and data analyses. All authors had read and approved the manuscript.

## Footnotes

1
https://statistics.eurotransplant.org/
